# Linking hIAPP misfolding and aggregation with type 2 diabetes mellitus: a structural perspective

**DOI:** 10.1042/BSR20211297

**Published:** 2022-05-18

**Authors:** Shahab Hassan, Kenneth White, Cassandra Terry

**Affiliations:** Molecular Systems for Health Research Group, School of Human Sciences, London Metropolitan University, London, United Kingdom

**Keywords:** amyloid, diabetes, hIAPP

## Abstract

There are over 40 identified human disorders that involve certain proteins folding incorrectly, accumulating in the body causing damage to cells and organs and causing disease. Type 2 Diabetes Mellitus (T2DM) is one of these protein misfolding disorders (PMDs) and involves human islet amyloid polypeptide (hIAPP) misfolding and accumulating in parts of the body, primarily in the pancreas, causing damage to islet cells and affecting glucose regulation. In this review, we have summarised our current understanding of what causes hIAPP to misfold, what conformations are found in different parts of the body with a particular focus on what is known about the structure of hIAPP and how this links to T2DM. Understanding the molecular basis behind these misfolding events is essential for understanding the role of hIAPP to develop better therapeutics since type 2 diabetes currently affects over 4.9 million people in the United Kingdom alone and is predicted to increase as our population ages.

## Introduction

### Type 2 diabetes is a complex disease

Diabetes mellitus is the name given to a group of metabolic diseases characterised by chronic hyperglycaemia as a result of defective glucose regulation. Symptoms of diabetes and the severity can vary depending on the type of diabetes. Diabetes mellitus can be split into type 1 and type 2 diabetes. In type 1 diabetes mellitus (T1DM, previously known as insulin-dependent diabetes or juvenile diabetes) the pancreas is unable to produce any insulin. Type 2 diabetes mellitus (T2DM) usually occurs in adults and is due to the pancreas being unable to produce fully functioning insulin in sufficient quantities. Defective insulin action and/or secretion results in metabolic abnormalities and the associated chronic hyperglycaemia results in long-term damage leading to organ failure. During the early stages of T2DM, patients can be asymptomatic. Symptoms of chronic hyperglycaemia are polyuria, polydipsia, weight loss, polyphagia, and blurred vision in addition to growth impairment and susceptibility to infection [1]. Long-term diabetes can lead to retinopathy, loss of vision, nephropathy, kidney failure, peripheral neuropathy, foot ulcers, amputations, and Charcot joint disease [2]. Also common in diabetic patients are hypertension and abnormalities in lipoprotein metabolism [3]. In addition to these complications, autonomic neuropathy can cause various disorders including genitourinary, cardiovascular, gastrointestinal complications, and sexual dysfunction [1]. Cancer is reportedly the current leading cause of death in diabetic patients in the United Kingdom [4] in addition to cerebro–cardiovascular, renal disease, and malignant neoplasms (particularly liver and pancreas neoplasms) [5]. The prevalence of diabetes is rising rapidly especially in low- to middle-income countries and is thought to be associated with obesity, poor access to medication, and infections [6]. There has been an increase in T2DM cases worldwide from 108 to 422 million between 1980 and 2014 [7] and cases are predicted to rise due to our ageing population and the rise in obesity.

Diabetes is a complex disease and is still not fully understood. Currently, there is no cure. Treatments simply alleviate the symptoms but do not stop the disease from progressing. Diabetes is a financial burden on health systems worldwide. Currently, in the United Kingdom alone, the cost for treatment, intervention, and complications are estimated to cost the National Health Service (NHS) £9.8 billion annually and is predicted to increase to £16.9 billion by 2035 [8]. More recently, evidence suggests that T2DM is strongly associated with other disorders such as Alzheimer’s disease (AD) and dementia [9,10]. Hence, there is an urgent need to better understand the disease at the molecular level to develop better treatments and/or preventative measures.

Proteinopathies or protein misfolding disorders, PMDs, are diseases resulting from incorrect protein folding, over 40 of which have now been identified [11]. Protein misfolding is the process where a protein does not fold into its correct three-dimensional structure resulting in loss of normal function and/or gain of toxicity. Misfolded proteins form proteinaceous aggregates that deposit in the body and cause severe damage to cells and the surrounding area. Aggregates are known to accumulate in the brain causing neurodegenerative disorders such as AD and Parkinson’s disease (PD) but can also damage other organs such as the pancreas in T2DM. Amyloid deposits from the pancreas of a T2DM patient were first described as hyaline lesions in 1901 [12] that were later described as proteinaceous amyloid plaques [13]. These deposits were shown to be composed of a neuropeptide consisting of 37 amino acids now commonly known as human islet amyloid polypeptide (hIAPP) or amylin [14–16]. In T2DM, it is these hIAPP deposits that are thought to deposit in the islet cells of the pancreas and as a result, disrupt normal glucose regulation thus causing T2DM. These hIAPP amyloid plaques have been used to distinguish between type 1 and type 2 diabetes since their presence is considered to be linked to the development of T2DM. Despite the considerable research linking hIAPP with type 2 diabetes [17,18], the molecular mechanisms explaining how hIAPP misfolds, deposits, and spreads to different parts of the body is still poorly understood, limiting our understanding of how hIAPP is linked to T2DM onset/progression.

In this review, we have reviewed the published structures of hIAPP in its different misfolded conformations (monomeric, oligomeric, and fibrillar) including mutagenic studies to try to understand how different parts of the protein and its different conformations are associated with disease. We have also summarised where these different conformations accumulate in the body and cause damage as it is now known that misfolded hIAPP accumulates in different parts of the body in addition to the pancreas. hIAPP has also been shown to interact with other amyloidogenic proteins accelerating their misfolding. This highlights the importance in understanding hIAPP not just to understand T2DM, but its role in related conditions such as AD and PD. Our review is novel since we have summarised all the mutations, all of the structures of hIAPP and locations of hIAPP deposits that have been published to date thus providing an up-to-date review that summarises what is known about hIAPP misfolding and aggregation and highlight gaps in our knowledge that urgently need addressing.

## The physiological function and structure of hIAPP

HIAPP is expressed in the pancreatic β-cells of the Islets of Langerhans and belongs to the calcitonin family of peptides. IAPP acts in an endocrine as well as auto and paracrine manner involved in the regulation of glucose, bone resorption [19], renin activity, vasodilation, and gastric emptying [20]. The gene encoding hIAPP is on chromosome 12 and has three exons. hIAPP is expressed as an 89-amino acid pre-pro-peptide. After translation, the 22-amino acid signal peptide is cleaved from the N-terminus to form a 67-amino acid propeptide (called ProIAPP). ProIAPP then enters the lumen of the endoplasmic reticulum (ER) where a disulphide bond between Cys^2^ and Cys^7^ is formed. It is then transported to the Golgi complex where it is packed into secretory granules and further cleaved to form a 37-amino acid peptide that is post-translationally modified by amidation at the C-terminal tyrosine. It is this active form that is co-secreted with insulin to regulate glucose levels in the body. IAPP is found in all mammals and healthy individuals and is co-produced with insulin in a molar ratio of 1:100 [21].

IAPP can undergo further post-translational modifications such as O-glycosylation [22] at Thr^6^ and Thr^9^ yet the physiological role of glycans and their role in disease remains unclear.

IAPP is normally soluble, monomeric, and natively unfolded in solution in its physiological form. It is described as an intrinsically disordered protein that has no regular three-dimensional structure (which is related to the presence of residues such as Ala, Asn, Gly, Ser, and Thr) and is positively charged at physiological pH [23]. IAPP is a highly conserved protein. There is 89 and 84% sequence conservation between hIAPP and the feline and rat protein respectively [24]. The sequence is highly conserved at the N-terminus (residues 1–18) with more variability between species at the C-terminus (residues 20–29) and in the pro-peptide region. Normally under physiological conditions, hIAPP is co-expressed, co-processed, and co-secreted with insulin [25–28]. Under hyperglycaemic conditions, hIAPP is overexpressed. In chronic hyperglycaemia, present in diabetics, an increase in IAPP:insulin ratio is usually observed [29].

## hIAPP has a high propensity to form amyloid

Human IAPP is a highly amyloidogenic peptide and there is extensive evidence demonstrating that it can adopt different conformational states. Changes to the local environment, or in particular, changes in hydrophobicity to residues 20–29 (known as the self-recognition element, SRE), are thought to be crucial for amyloid formation [30]. Sequence diversity within regions 20–29 corresponds to whether IAPP forms amyloid which corresponds directly with the ability of certain species to develop spontaneous T2DM such as humans, primates, and felines that all have sequences thought to be amyloidogenic. Species such as rodents have three proline residues in the IAPP sequence instead of Ala^25^, Ser^28^, Ser^29^, and do not form amyloid or T2DM. The S20G mutation which is highly amyloidogenic has been associated with early-onset T2DM and has been found in the Japanese and Chinese populations [31]. IAPP is largely unfolded apart from residues 8–16 which are amphipathic and typical of a membrane-bound helix that can potentially interact with membranes [32]. In T2DM, hIAPP is thought to interact with membranes, and in doing so, adopts a more α-helical structure [33]. This interaction with membranes is thought to initiate the aggregation of hIAPP and the misfolding cascade that is associated with T2DM.

## The link between misfolded hIAPP and type 2 diabetes

This ability of hIAPP to form amyloid like other PMDs and the observation that over 90% of T2DM patients have hIAPP deposits in the form of extracellular fibrillar aggregates in their pancreas [18] strongly suggests hIAPP as an important factor in the progression of T2DM. The first reported observations of hIAPP deposits in post-mortem pancreatic parenchymal cells from T2DM patients dates back to 1901 [12]. Many years later, these deposits were isolated and identified as a 37-amino acid peptide that was named IAPP [15,16]. These amyloid deposits have been associated with the progression of T2DM for some time, however exactly how they are associated with disease is still an area of debate. In primate models, the formation of these aggregates occurs before clinical symptoms such as altered glucose metabolism [34], however it is unclear if this is the case in humans.

Amyloid can be defined as fibrillar deposits of proteins that contain a β-sheet structure and as a result, have enhanced dye-binding capacity to Thioflavin T [35], Congo Red [36], or staining with iodine [37]. Amyloid fibrils tend to be unbranched, several micrometres in length, are usually twisted and have a ‘cross β’ three-dimensional structure being composed of β-strands arranged perpendicular to the fibril axis held together via hydrogen bonds [38].

**Figure 1:**
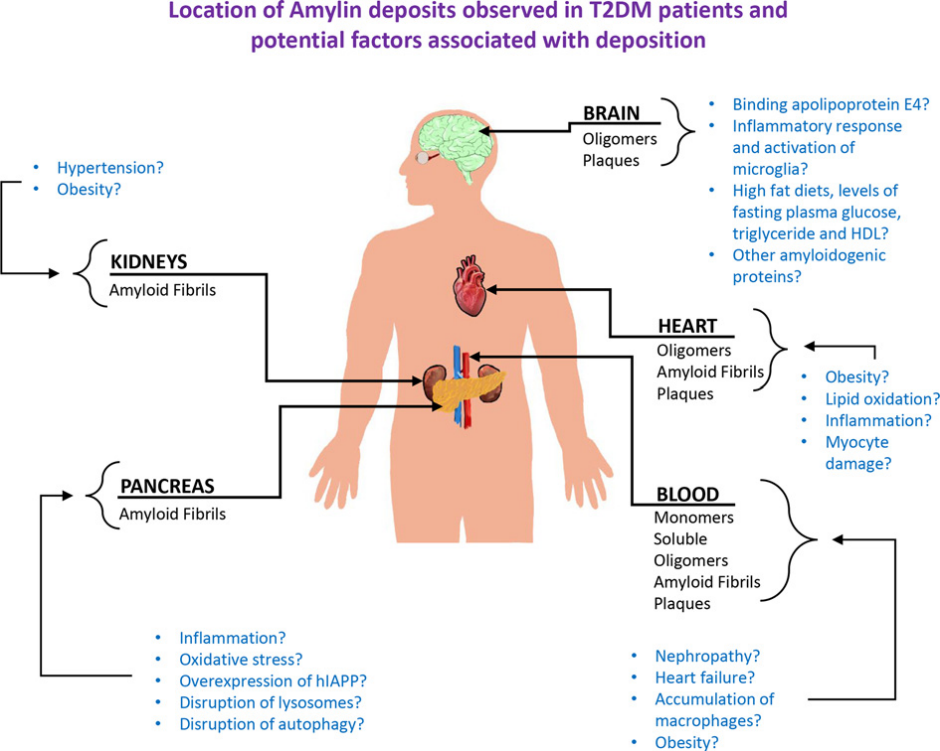
Diagram summarising the location of hIAPP that has been identified in different parts of the body in T2DM patients The conformations of hIAPP that have been identified in studies to date have been highlighted. Also highlighted are factors thought to contribute to this protein deposition in these different parts of the body summarising what has been suggested in the literature.

In addition to being observed in the pancreas, misfolded forms of hIAPP have also been identified in different organs in T2DM patients (Table 1 and Figure 1), including the kidneys [39], the heart [40], brain [41] plus circulating in the blood [41] and perivascular spaces [42]. It has been suggested that hIAPP aggregates, like prions, can spread to other parts of the body in a prion-like mechanism [43], however direct evidence for this is limited.

**Table 1:** Studies that have observed accumulation of misfolded hIAPP in T2DM patients in comparison to healthy controls

Tissue/organ	Methods used	Observations	Reference
Blood	Western blotting and ELISA	Monomers in plasma, white blood cells and red blood cells. hIAPP-coated RBCs show reduced functional haemoglobin and have lower deformability	[44]
Blood	Western blotting, TEM, CD	Soluble oligomers in the form of homooligomers (trimers, hexamers, dodecamers) and heterooligomers varying in size (1–400 nm) from sera of children with T1DM and T2DM and obese children. Fibrils 55–252 nm in length present in T2DM and obese patients	[45]
Blood	Immunochemistry with anti-IAPP antibodies, Congo Red staining	hIAPP plaques, amyloid fibrils found in the blood vessel walls and also perivascular spaces	[41]
Brain	Immunohistochemistry with anti-IAPP antibodies, Congo Red staining, Western blotting, ELISA, qRT-PCR	hIAPP oligomers (trimers 12 kDa, tetramers 16 kDa, and pentamers 20 kDa) and hIAPP plaques > 20 µm in diameter identified in the temporal lobe grey matter. Tetramers abundant in brain homogenates. Tissues showed increased interstitial space, vacuolation, spongiform change, and capillaries bent at hIAPP accumulation sites. hIAPP mRNA in the brain is ∼10^4^ lower than in the pancreas	[41]
Heart	Immunohistochemistry and Western blotting	Oligomers (>32 kDa), fibrillar tangles, plaques, found in diabetic and obese patients’ hearts. Small oligomers found in healthy hearts from obese patients	[40]
Kidneys	Immunohistochemistry, Immunogold staining TEM	hIAPP deposits composed of straight non-branching fibrils 12–16 nm in diameter observed in Kimmelstiel–Wilson nodules, Bowman’s capsule in patients with diabetic nephropathy	[39]
Pancreas	Immunohistochemistry with anti-IAPP antibodies, qRT-PCR	Fibrillar deposits in pancreatic islets	[41]
Pancreas	Congo Red staining	Fibrillar deposits in pancreatic islets adjacent to the islet cells	[46]
Pancreas	Peroxidase-antiperoxidase staining, Immunohistochemistry, Congo Red staining	Amyloid deposits plus amorphous deposits stained with Congo Red in Islets of Langerhans. Amyloid did not stain with anti-insulin antiserum	[18]
Pancreas	Congo Red staining	Amyloid fibrils found mostly between capillaries and epithelial islet cells. Amyloid likely originated from β-cells: cells show amyloid-filled invagination and orientation of hIAPP fibrils were seen perpendicular to cell surface. Enlarged macrophages with amyloid deposits. β-cells degraded in the islets	[47]
Pancreas	Microscopy staining with Haematoxylin, Eosin, Picric acid stain	Deposition of ‘hyaline material’ in pancreatic parenchymal cells and immediately outside the walls of the capillaries	[12]

Summarised here are the location of the misfolded hIAPP, the morphology of the misfolded forms, and experimental techniques used to analyse the misfolded aggregates from tissues acquired post mortem. Abbreviations: CD, circular dichroism; ELISA, enzyme-linked immunosorbent assay; qRT-PCR, quantitative real-time polymerase chain reaction; TEM, transmission electron microscopy.

## The link between hIAPP misfolding and pathology

The likely event causing hyperglycaemia in T2DM is dysfunction or loss of β-cells leading to reduced secretion of insulin and development of insulin resistance [48]. Here we consider the role that IAPP aggregation may have in contributing towards β-cell loss or dysfunction, and the mechanisms that may be involved. The first discoveries of amyloid deposits in pancreatic islets were made using tissues recovered post-mortem [18], making it difficult to establish causality between IAPP aggregation and β-cell dysfunction *in vivo*. The use of transgenic animal models has provided a longitudinal approach to support the correlation between IAPP aggregation and β-cell loss. Mice and rat IAPP do not aggregate, and these rodents do not normally develop diabetes. Animals where the human IAPP gene is highly expressed, develop diabetes as they grow during their lifetime [49]. The HIP rat model (rats transgenic for human IAPP) is an exemplar that recapitulates the development of diabetes starting from a non-diabetic state at 2 months, impaired fasting glucose at 5 months, and diabetes at 10 months [50]. At 2 months of age there are signs of apoptosis induced in β-cells by aggregates of IAPP, at 5 months there is a 50% loss of β-cells rising to 70% at 10 months, observations that have also been identified in humans [51]. Further studies in humans have confirmed this relationship between IAPP amyloid formation, β-cell loss, and β-cell apoptosis [52].

*In vitro*, hIAPP has a strong tendency to aggregate and will do so spontaneously within hours at µM concentrations [53]. *In vivo*, IAPP is stored in secretory granules at mM concentrations [54] but a combination of being in a solid–liquid phase, in the presence of Zn^2+^ and insulin at a slightly acidic pH prevents aggregation, especially during assembly of granule contents [55,56]. The proteostatic environment is normally maintained when granules release their contents after secretion, but when there is a higher demand for insulin, local concentrations of IAPP will be higher and there is a greater chance of aggregation on and around the β-cell after release. Circulating levels of IAPP are reported to be ∼10 pM [49] and IAPP carries a positive charge at neutral pH and can bind to membranes or lipid components carrying a negative charge both *in vitro* and *in vivo*. Interaction with membranes can trigger a change in IAPP structure from the natural unfolded state to an α-helical structure that can insert into membranes [57,58] or form toxic oligomers [59]. Studies using cultured islet cells have shown that such IAPP oligomers can induce apoptosis [60], and *in vivo* studies have identified oligomeric structures at the site of β-cell loss in pancreatic islets [59].

There are several factors that could disturb proteostasis and induce toxic aggregation of IAPP inside β-cells. Intracellular toxic aggregates of IAPP were identified in biopsies of islets from patients with T2DM with a perinuclear or cytosolic distribution [61], the latter being associated with mitochondrial damage since cytosolic oligomers are thought to directly bind and disrupt mitochondrial membranes. There is also evidence of damage to membranes in the secretory pathway. Conditions in which proteostasis is disrupted can cause intracellular aggregation of IAPP resulting in deposits of oligomers in the secretory pathway and granules. These can include overexpression [61], which can lead to increased cytosolic Ca^2+^ activation of calpain and apoptosis [62], disruption of lysosomes and hence proteostasis [63] and disruption of autophagy [64,65].

Overexpression of IAPP could activate the unfolded protein response (UPR). A series of three signalling pathways in the ER are triggered by IAPP protein misfolding and result in a local inflammatory response involving the release of pro-inflammatory cytokines IL-6, IL-8, MCP-1, and TNFα [66]. MCP-1 has been shown to increase the expression of IAPP in a mouse cell line [67]. Of the three pathways, the IRE1 pathway is prominent with high expression in the β-cell [68]. All three pathways IRF1, PERK, and ATF6 converge to activate c-Jun N-terminal kinases (JNKs) and the key inflammatory regulator NF-kB. The cytokines can induce insulin resistance in β-cells and peripheral tissues and could activate the NLRP3 inflammasome leading to secretion of IL-1β. Treatment of islets with physiological concentrations of IL-1β has been shown to induce IAPP aggregation [69]. hIAPP aggregates have been shown to elicit inflammatory responses in β-cells directly or by recruiting macrophages [70–72].

Larger amounts of oligomers have been found in β-cells of obese T2DM patients compared with non-obese patients, suggesting that other environmental factors such as oxidative stress can affect aggregation. Chronic hyperglycaemia or dyslipidaemia prevalent in T2DM will increase the stress responses [66]. The β-cells are particularly sensitive to oxidative stress, hence any oxidative insults for example, through excessive mitochondrial activity in hyperglycaemia will exacerbate stress responses [66] causing damage or death to β-cells.

### hIAPP deposition in the brain

A number of studies have explored the link between diabetes and neurological pathology mediated via IAPP. One of the first reports of hIAPP deposition in the brain reported that levels of oligomeric hIAPP were highest in brain homogenates taken from T2DM patients with dementia, compared with AD patients and controls [41]. They also showed that brain homogenates from those with T2DM and dementia contained ten-times the amount of hIAPP oligomers compared with the other groups and also identified hIAPP plaques in these patients. They also quantified the amount of hIAPP mRNA in the brain and reported that hIAPP mRNA in the brain is ∼10^4^ lower than in the pancreas. These findings were corroborated using HIP rat models where hIAPP is expressed only from the pancreas [73] demonstrating that IAPP is produced in the pancreas and moves to the brain. These transgenic hIAPP rats spontaneously develop a diabetic state by 10–12 months and have the advantage of demonstrating a prediabetic state at age 8–10 months, characterised by high levels of circulating insulin and hIAPP [73]. Loss of brain function was observed (in the T2DM state) and was associated with the deposition of hIAPP plaques in the pancreas and brain. hIAPP deposition in the brain can trigger an inflammatory response and activation of microglia. hIAPP deposition in blood vessels of the brain has previously been shown to cause endothelial disruption. In the rat model hIAPP deposition can be increased by co-binding of apolipoprotein E4, and likely other apolipoproteins, leading to increased vascular pathology [74].

A study using brain samples from 2365 patients with and without diabetes, assessed AD neuropathology and concluded that diabetes is not associated with AD pathology [75]. Diffuse plaques (plus other markers of AD) were not significant in those with diabetes but there were increased brain infarcts (e.g. Lacunes) suggesting that diabetes is associated with cerebrovascular neuropathology but not AD. However, since diabetes was not determined clinically (patients self-diagnosed their condition) and patients were not separated into type 1 and type 2, it is difficult to link these results specifically to T2DM.

There are, however, several studies that have shown a link between T2DM and AD. hIAPP deposits were found in brain blood vessels and associated parenchyma in patients with T2DM and AD some of which also included amyloid-β (Aβ). Interestingly, there were also hIAPP deposits in the AD group who had no apparent diabetes. The data suggested a model in which pancreatic hIAPP creates deposits in brain tissues where the initial aggregation events may be triggered by the high levels of hIAPP that occur in the prediabetic state. The results also demonstrate the potential for co-aggregation with other amyloidogenic proteins potentially influencing amyloid formation. These observations have been confirmed in other studies using animal models. Since rodent IAPP does not aggregate, this property is exploited in transgenic mice and rats expressing the aggregating human form of IAPP. A study using transgenic hIAPP mice focussed on the ability of α-synuclein to enhance amyloid formation involving co-aggregation with hIAPP in islets [76] using both endogenously and exogenously expressed α-synuclein. Analysis of fibrils isolated post-mortem from T2DM islets revealed the presence of α-synuclein as well as IAPP [76]. The association of α-synuclein and IAPP in the pancreas and the brain has also been found in primates (cynomolgus monkeys) that spontaneously developed T2DM [77]. Amyloid deposits were also found in the brains of transgenic hIAPP mice fed a high-fat diet [78]. The ability of well-characterised amyloid proteins to co-aggregate with hIAPP has been demonstrated *in vitro* for Aβ 42 [79], and α-synuclein [76].

Further work has focussed on the potential role of other factors that could modulate IAPP aggregation, in particular obesity or the pre-diabetic state. A positive correlation between brain amyloid deposits and levels of fasting plasma glucose, triglyceride, and HDL has been observed in cynomolgus monkeys [77] and in mouse hIAPP models where high-fat diets induced larger amounts of amyloid deposits in the brain [78].

### Presence of hIAPP deposits in the kidney

Diabetes and hypertension, individually or combined are thought to account for 80% of all cases of end-stage renal disease. Nephropathy caused by diabetes is due to damage to blood vessels which leads to chronic kidney disease. A single study on humans has described deposits of IAPP in kidneys of patients with diabetic nephropathy (DN) [39]. hIAPP deposits were identified in ∼50% of patients with DN but not in patients with other kidney pathologies. A small number of samples from obese patients also contained hIAPP deposited in glomeruli (mesangial cells), blood vessels and the interstitium and fibrillar deposits were identified in the mesangium. Notably, the glomerular filtration rate was significantly reduced by ∼30% in patients with hIAPP deposits compared with those with DN without deposits. The same study demonstrated the ability of fibrillar hIAPP to induce apoptosis in primary human mesangial cell cultures [80]. In the HIP rat model (but not the UCD-T2DM rat model) hIAPP deposits were found in arterioles, interstitial tissue between tubules and glomeruli [44]. Mice that received hIAPP developed impaired kidney function, assessed by excretion of urinary protein and structural damage to the kidney in the form of increased fibrosis of the glomeruli [81].

### hIAPP deposits in the blood

hIAPP monomers have been detected in the plasma, white blood cells, and red blood cells in blood from a T2DM patient with nephropathy [44]. In a comparative study between patients with T2DM, heart failure or both, the highest levels of hIAPP were detected in red blood cells of patients with T2DM and heart failure [44]. Further studies using the HIP rat models showed higher levels of IAPP in red blood cells compared with control rats with red blood cells having lower deformity but being less concave in shape (more spherical) yet had normal oxygen (O_2_) binding [44]. hIAPP deposits found in the kidneys of HIP rats showed vascular hIAPP deposition that correlated with the accumulation of macrophages [44]. Elevated erythropoietin (EPO) and accumulation of hypoxia-inducible factor (HIFs) in the kidneys is thought to be a direct response to hIAPP-coated red blood cells.

Larger conformers of misfolded hIAPP have also been identified in the blood of T2DM patients. Oligomers in the form of homodimers (trimers, hexamers, dodecamers) and heterooligomers varying in size (1–400 nm) have been identified from sera of children with T2DM that were also present in obese children and those with T1DM [45]. Fibrils and hIAPP plaques have been found in the blood vessel walls and perivascular spaces of T2DM patients [41,45]. Fibrils were reported to be 55–252 nm in length and were present in T2DM and obese patients but not healthy controls [45].

### IAPP and cardiovascular disease

Diabetes is known to be a risk factor for cardiovascular disease, attributed to the damage caused by high-glucose levels on vasculature supplying the heart. Here we consider whether IAPP could contribute to the risk of heart disease for diabetics by directly affecting the function of cardiomyocytes and surrounding cells. There are a very limited number of studies that have investigated IAPP aggregation in human heart tissue. Despa et al. [40] found IAPP oligomers, fibrils, and plaques in failing hearts of diabetics and obese patients but not in non-failing hearts of diabetics or obese patients or normal controls. Analysis of heart tissue from transplant patients who later became diabetic, and were considered prediabetic, revealed oligomeric forms of hIAPP attached to the sarcolemma or inside myocytes [82]. Immunohistochemistry revealed hIAPP in blood vessel walls, perivascular space, sarcolemma and staining with Congo Red indicated deposition of amyloid structures between myocytes. The pattern of staining is consistent with hIAPP binding to membranes then entering parenchyma, albeit by unknown mechanisms.

In a follow-up study [83] oligomeric amylin was also found in the blood, pancreas, and heart of obese, heart failure patients who were not diabetic. Levels of 4-hydroxynonenal (HNE) and malondialdehyde (MDA) (lipid breakdown) adducts of amylin were measured as markers of oxidative damage in extracts of left ventricles and were found to be higher in obese or diabetic patients with heart failure compared with controls. The same groups had increased expression of IL-1β compared with controls.

These findings in patients suggest a correlation between IAPP aggregation, lipid oxidation, inflammatory stress, and myocyte damage, which was investigated using rat models and cultures of cardiomyocytes [40]. Treatment of rat cardiomyocytes with 50 µM hIAPP, but not rat IAPP, induced changes in intracellular calcium, attributed to increased sarcolemma permeability. Monomeric, dimeric, and trimeric oligomers of hIAPP could be identified by Western blotting and deposits were found on sarcolemma membranes by fluorescence microscopy. Heart tissues were examined in the HIP-rat model in the pre-diabetic state and hIAPP was found in blood vessel walls and parenchyma of left ventricles in the pre-diabetic state [82].

HIP rats were compared with the UCD-T2M rat model, which is not transgenic for hIAPP and in which diabetes develops due to β-cell dysfunction [40]. In the pre-diabetic state, hIAPP deposits were found in hearts of HIP, but not UCD-T2M, rats with concomitant compromise of Ca^2+^ movements in HIP cardiomyocytes not found in UCD-T2M myocytes. Prediabetic HIP rats show signs of cardiac abnormality, with accumulation hIAPP, whereas UCD-T2M prediabetic rats do not.

A model of the disruption of Ca^2+^ movements in myocytes by hIAPP suggests an initial attachment to the sarcolemma, and then the formation of oligomers that could create cation-selective channels in the sarcolemma and also develop into fibrils. The membrane-attached fibrils could amplify structural alteration of membrane and hence Ca^2+^ dysregulation, leading to remodelling of myocytes via a Ca^2+^-dependent activation of HDAC NFAT transcription factors [84].

Liu et al. [83] further investigated the role of inflammation in the HIP-rat model. Lipid oxidation was associated with aggregation of hIAPP and formation of hIAPP HNE– and MDA–lipid oxidation adducts. There was also an increase in IL-1β and TNFα but not IL-6 or IL-10 in the HIP model. A role of the NLRP3 inflammasome was suggested. Rat hearts perfused for 2 h with 50 µM hIAPP had increased ROS and IL-1β, which was prevented by co-administration of 50 µM poloxamer 188 surfactant to prevent amylin aggregation and binding or 5 mM N-acetyl cysteine to prevent lipid peroxidation.

Similar results have been found in mice perfused with aggregated hIAPP, which induced formation of hIAPP HNE– and MDA–adducts in the heart and activation of IL-1β. In a mouse knock-out model which does not express endogenous IAPP [85], administration of exogenous hIAPP led to deposits of hIAPP aggregates on the sarcolemma and changes in myocyte Ca^2+^ signalling. The model was advantageous since other aspects of diabetes pathology such as hyperglycaemia or cardiac remodelling found in diabetes would be absent.

These studies suggest that hIAPP can bind to cardiomyocytes, aggregate, and induce stress but it also seems possible that accumulation in vasculature of the heart could also contribute to compromising heart function.

### hIAPP deposition in the eye

IAPP has been found in the eye of individuals with AD [86,87]. It is assumed that levels of hIAPP are higher in the retina of those with T2DM, however, we could not find any reports of this which is surprising since retinopathy caused by damage to blood vessels, affects one-third of all people with diabetes and can lead to macular oedema, glaucoma, and cataract formation. Retinal and hippocampal levels of high molecular weight IAPP have been shown to correlate with retinal levels of Aβ42 [86,87]. Studies looking at IAPP levels in the eye are limited and characterisation of the different conformations of hIAPP found in T2DM patients and their association with other amyloidogenic proteins known to occur in the eye (such as crystallin proteins associated with cataract formation) are needed to see if there is a link.

## The misfolding of IAPP involves different protein conformations

Most of our information regarding the misfolding pathways come from *in vitro* studies so the exact mechanism of misfolding *in vivo* remains unclear. IAPP amyloid formation is thought to occur via a nucleation-dependent polymerisation mechanism *in vitro* [88]. Evidence suggests that IAPP oligomerises intracellularly (perhaps as a result of incorrect proIAPP processing) in the islet β-cell secretory granules and are released from these granules and deposit into the extracellular space when β-cells degenerate [52].

The three main stages involved in amyloid formation are thought to include: (1) primary nucleation (lag phase) where small soluble oligomers are formed from monomeric peptides followed by (2) elongation (log phase) involving the formation of protofibrils, and fibril growth followed by (3) the plateau (or saturation) phase where mature amyloid fibrils dominate. hIAPP amyloid fibrils are further stabilised by their interactions with proteins such as serum amyloid P, apolipoprotein E, and perlecan [89]. Perlecan is thought to provide further stability to IAPP fibrils by assisting binding to membranes of islet capillaries. Like other amyloidogenic proteins, hIAPP amyloids are stable structures that are resistant to proteolysis [90] and hence can accumulate easily within the body and are difficult to remove. The stability of IAPP (28−33) polar fibrils is attributed to van der Waals and electrostatic bonds including hydrogen bonds [91]. Once formed, the fibrils themselves can then act as a catalytic surface to nucleate the formation of new aggregates from monomers in a process known as secondary nucleation [53].

Glycosaminoglycans such as heparan sulphate, are negatively charged polymers found in the extracellular matrix or on the membrane surface of cells. They have been found associated with hIAPP deposits in T2DM and are thought to be essential for amyloid formation [92]. Lipid membranes themselves have also been suggested as a surface from which nucleation and polymerisation of hIAPP can occur in a two-step process by stabilising and catalysing the aggregation process [93,94]. Pre-fibrillar hIAPP (via the N-terminus, residues 1–19) binds to negatively charged membranes (located on the cytosolic side of the plasma membrane) via electrostatic interactions increasing the local concentration and a change in conformation (from a monomer in a random coil conformation to a transient α-helical form in the bound state to a β-sheet conformer in the form of an oligomer). This is thought to be followed by a second phase that involves the growth of the fibril on the membrane.

Like many PMDs, there is still debate over what conformation of the amyloidogenic protein is associated with disease. Since fibrillar amyloid deposits of hIAPP have been observed in islet cells in the majority of T2DM patients [18], it is thought that fibrils are directly associated with T2DM. These fibrils could contribute to T2DM by inducing cell membrane damage [95], directly damaging cells [96], or disrupting the extracellular matrix impairing cell communication [21]. Perhaps the fibrils are too large or too insoluble to have an active role in T2DM pathology and hence some researchers suggest that smaller oligomers (made prior to amyloid) produced intracellularly, can permeate the membrane and are responsible for β-cell toxicity [60,61].

Oligomers ranging from dimers to hexamers have been identified during the early stages of aggregation [97] and in particular dimers have been suggested as being particularly abundant. Studying such small assemblies, like in other PMDs, has proved difficult. This is due to them being transient, being present for only a short period of time before being converted into protofibrils. In addition, they are thought to be present in low concentrations hence better isolation techniques are needed to characterise them [98]. Oligomerisation of hIAPP into dimers and trimers has been associated with residues His^18^ and Tyr^37^ [99]. Residues 23–27 have been shown to be important in the structural rearrangement of random coil structures to pentameric β-sheet oligomers driven by hydrophobic and aromatic interactions between amyloidogenic regions 15–20 and 22–29 [100]. Molecular dynamic simulations revealed that oligomers have more predominantly α-helical structures; however, a subset of β-rich oligomers can adopt a β-barrel conformation thought to be the toxic oligomer species [101]. *In vitro* experiments suggest that oligomers contribute to toxicity by disrupting cell membranes by forming pores that allow too many Ca^2+^ ions to enter that can lead to apoptosis [24]. More evidence is needed to corroborate these findings *in vivo* including studying their structure in more detail (if indeed they exist *in vivo*) to understand how they function in disease. Two-dimensional infrared spectroscopy experiments have revealed that an oligomeric intermediate form of hIAPP consists of parallel β-sheets [102]. Interestingly, since the region between 20 and 29 in the amyloidogenic core (SRE) has been associated with fibril formation, it was thought that this core region formed β-sheets within the mature fibril however instead it showed a partially disordered loop/turn suggesting the intermediate has a transient β-sheet that forms during the lag phase of amyloidosis.

Recent *in vitro* experiments suggest that fibrillisation of 1–37 is a synchronous process where protofibril elongation and mature fibril formation occur simultaneously [103] rather than in discrete stages as reported for other amyloidogenic proteins such as Aβ [104]. These solution atomic force microscopy (AFM) experiments suggest that fibril elongation occurs from one end of the fibril only, however *in vivo* experiments are needed to corroborate these findings.

### Misfolding of other proteins associated with type 2 diabetes

Whilst there is ample evidence linking hIAPP with T2DM, there is some evidence in the literature suggesting other proteins may misfold or somehow contribute to the misfolding of other proteins involved in T2DM. ProIAPP also appears to misfold since amyloid deposits composed of fibrillar aggregates containing proIAPP have been observed in secretory granules in some β-cells in human IAPP transgenic mice [105].

Insulin degradation enzyme (IDE), which also degrades hIAPP [106], appears to also contribute to T2DM although there is no direct evidence showing that it misfolds. Evidence shows that IDE levels are greatly reduced (a reduction of ∼40%) in β-cells of T2DM individuals compared with non-diabetic controls [107]. The addition of Bacitracin, a chemical that inhibits IDE activity, results in the accumulation of IAPP and cytotoxicity in β-cell in cell models (using RIN-m5F cells; [108]) suggesting a link to T2DM.

Familial mutations in ubiquitin carboxyl-terminal hydrolase L1 (UCH-L1) are associated with PD where the variant I93M is found to aggregate [109]. UCH-L1 may also play a role in T2DM. Increased levels of hIAPP can down-regulate UCH-L1 which results in β-cell apoptosis via ER stress and reduced UCH-L1 levels have been observed in β-cells of T2DM patients [110]. This suggests that UCH-L1 may misfold resulting in ER stress-associated apoptosis, however further experiments are needed to understand the molecular details linking UCH-L1 with T2DM.

Aβ and α-synuclein, proteins that misfold in AD and PD respectively have been associated with IAPP misfolding and T2DM. *In vivo* studies show that intravenous injection of Aβ fibrils into hIAPP transgenic mice cause increased amyloid formation in islet cells [111] and co-expression of IAPP and Aβ in *Drosophila melanogaster* models result in co-deposition of amyloid peptides [112]. A significant increase (100%) in α-synuclein levels was detected in β-cells of T2DM patients compared with controls (non-diabetics) with very low (or undetectable) levels of monomeric α-synuclein reported [107].

## Our current understanding of hIAPP structures using high-resolution techniques

Understanding the structures of hIAPP assemblies is crucial for understanding exactly how IAPP is linked to T2DM. There are now many hIAPP structures that have been solved (Table 2) many to high resolution, including fibrils [103,113–127], monomers [32,128,129], oligomers (tetramers) [130], hIAPP analogues [131], hIAPP in combination with other proteins [117,132–134] and structures of pro-IAPP polypeptide [135], and give some insight into how hIAPP might be linked to T2DM. Some of these structures have been shown to be toxic to pancreatic cells such as fibrils [120] and oligomers [136] linking them to disease.

**Table 2:** Summary of all hIAPP protein structures solved to date including sample details, a brief description of the structures obtained, and the associated references in reverse chronological order by article publication date

IAPP sample preparation	Structural methods	Structural observations	PDB code	Reference
Synthetic hIAPP peptides seeded with *ex-vivo* T2DM patient-derived fibrils	Cryo-EM	Polymorph 1. Twisted, heterotypic fibrils, composed of two non-identical protofilaments with 589 Å repeating pitch. Cross-β scaffold with β-strands stacked with 4.8-Å spacing Two different core folds: Chain A core fold F15-S28 and Chain B core fold S20-G33. 3.8 Å resolution	7M61	[123]
Synthetic hIAPP peptides seeded with *ex-vivo* T2DM patient-derived fibrils	Cryo-EM	Polymorph 2. Twisted fibrils composed of two identical protofilaments with 547 Å repeating pitch. Cross-β scaffold with β-strands stacked with 4.8-Å spacing. The two protofilaments are related by a C2 symmetry axis with an extended core. Tyr^37^ forms a hydrogen bond promoting amyloidosis. 3.9 Å resolution	7M62	[123]
Synthetic hIAPP peptides seeded with *ex-vivo* T2DM patient-derived fibrils	Cryo-EM	Polymorph 3. Twisted fibrils composed of two identical protofilaments with 259 Å repeating pitch. Cross-β scaffold with β-strands stacked with 4.8-Å spacing. Two S-shaped, intertwined protofilaments same as 6Y1A. Two identical core folds for chain A and B from F15-S28. Tyr^37^ forms a hydrogen bond promoting amyloidosis. 4 Å resolution	7M64	[123]
Synthetic hIAPP peptides seeded with *ex-vivo* T2DM patient-derived fibrils	Cryo-EM	Polymorph 4. Twisted, heterotypic fibrils, composed of two non-identical protofilaments 589 Å repeating pitch. Cross-β scaffold with β-strands stacked with 4.8-Å spacing. Two different core folds: Chain A core fold F15-S28 and Chain B core fold S20-G33. Tyr^37^ forms a hydrogen bond promoting amyloidosis. 4.1 Å resolution	7M65	[123]
Human, IAPP analogue (AM833 / Cagrilintide) mutations N14E, V17R, A25P, S28P, S29P, Y37P analogue fusion with MBP	X-ray diffraction	Cys^7^–Cys^6^ disulphide bond, α-helix from 5 to 18, and S34-P37 (SNTP) disordered at C-terminal end. 2.89 Å resolution	7BG0	[131]
Human, full length expressed in *Escherichia coli*	Cryo-EM	Fibrils composed of two symmetrically related protofilaments with ordered residues 14–37. 3.4 Å resolution	6VW2	[122]
Human pro-IAPP in DPC micelles at pH 4.5	Solution NMR	Open conformer with average angle between α1 and α2 ranged from 162 to 170°. ProIAPP is a dynamic molecule with four α-helices. The first two within the mature IAPP sequence, the second two form part of the C-terminal prohormone segment (Cpro)	6UCJ	[135]
Human pro-IAPP in DPC micelles at pH 4.5	Solution NMR	Bent conformer with average angle between α1 and α2 ranged from 119 to 127°. ProIAPP is a dynamic molecule with four α-helices. The first two within the mature IAPP sequence, the second two form part of the C-terminal prohormone segment (Cpro)	6UCK	[135]
Human, wildtype, residues 14–37	Cryo-EM	WT has fibrils with an ordered core composed of two-protofilament amyloid structures composed of two S-shaped subunits, with 25 nm crossover lengths. Left hand helical twist. 3.6 Å resolution	6ZRF	[124]
Human, S20G early-onset variant with amidated C-terminus	Cryo-EM	S20G fibrils consist of two types. ‘2PF’ has two protofilaments in the fibril. Approximately 76% of fibril segments with a 50-nm repeat. Left hand helical twist. 4.0 Å resolution	6ZRQ	[124]
Human, S20G early-onset variant with amidated C-terminus	Cryo-EM	S20G fibrils consist of two types. ‘3PF’ has three protofilaments in the fibril. Approximately 24% of fibril segments have a 50-nm repeat. Left hand helical twist. 3.9 Å resolution	6ZRR	[124]
Human, full length synthetic hIAPP pH 6, amidated with Cys^2^–Cys^7^ disulphide bond	Cryo-EM	Three amyloid fibril polymorphs formed *in vitro* Polymorph 1 (90% of all fibrils), right-handed helical symmetry, 2.5–4.5 nm wide, have two S-shaped, intertwined protofilaments, 4.2Å resolution. Polymorph 2 composed of two protofilaments (10% of all fibrils) 17–52Å width, <4 Å resolution. Polymorph 3 composed of two protofilaments (<1 of all fibrils) 110 Å wide, 8 Å resolution	6Y1A	[125]
Human IAPP 1–37 polypeptide	Cryo-electron tomography, Negative stain and cryo-EM	Fibrils 12 nm in width composed of two protofilaments (6 nm in width) intertwine to form fibrils several micrometres long	N/A	[103]
Human IAPP segment 15-FLVHSSNNFGA-25 m wildtype	Electron crystallography and Micro ED	Fibrils composed of tightly mated curved pairs of parallel in-register β-sheets, cytotoxic. 1.4 Å resolution	5KO0	[120]
Human IAPP 19-SGNNFGAILSS-29 with early-onset S20G mutation	Electron crystallography/Micro ED	Fibrils formed of pairs of parallel in-register curved β-sheets. Shares features with full-length hIAPP fibrils, also toxic. 1.9 Å resolution	5KNZ	[120]
11–17 hIAPP peptide (RLANFLV)	X-ray crystallography	Tetramer composed of 4 β-sheets that pack to form a tetramer. Consist of hydrogen-bonded dimers (composed of antiparallel β-sheets) that are held together by hydrophobic interactions. 1.8 Å resolution	5UHR	[130]
Human, full length, oxidised and amidated hIAPP	Solution NMR	Monomeric hIAPP in water. N-terminus of oxidised peptide forms an α-helical structure lacking in the reduced state. Disulphide bridge in oxidised hIAPP stabilises an α-helical structure at the N-terminus preventing the peptide from aggregating	5MGQ	[129]
Human IAPP, residues 10–30, in complex with an aggregation inhibitor HI18	Solution NMR	Monomeric IAPP adopts a β-hairpin conformation upon binding to H18. The β-hairpin is wrapped by the two HI18 subunits, each contributing two α-helices and a short β-strand that extends the IAPP β-hairpin, giving rise to a four-stranded intermolecular antiparallel β-sheet	5K5G	[134]
Human IAPP peptide residues 13–18	X-ray crystallography	IAPP segments from fibril core. 13-ANFLVH-18, form parallel, in-register β-sheets. 1.61 Å resolution	5E5X	[119]
Human IAPP peptide residues 16–21	X-ray crystallography	Hexameric segment 16-LVHSSN-21, forms a staggered in-register steric zipper. 1.66 Å resolution	5E5Z	[119]
Human IAPP peptide residues 23–29	X-ray crystallography	23-FGAILSS-29, steric zipper with β-strands arranged anti-parallel in a β-sheet and the two mating sheets running parallel to each other. 1.79 Å resolution	5E61	[119]
Human IAPP peptide residues 22–28	X-ray crystallography	22-NFGAILS-28 forms an out-of-register steric zipper. 1.24 Å resolution	5E5V	[119]
Human full-length synthetic hIAPP peptide	EPR, TEM and computer modelling	Fibrils composed of two β-strands, forming two opposing β-sheets that wrap around one another, with hydrophobic fibril ends. Fibrils have a left-handed helical twist and are composed of staggered hIAPP monomers	N/A	[126]
Human full length hIAPP, with Cys^2^–Cys^7^ disulphide bond and amidated at C-terminus, pH 7.3 in SDS micelles	Solution NMR	Amidated, monomeric hIAPP in membrane environment. Ordered C-terminus, kinked helix motif, with residues 7–17 and 21–28 in a helical conformation. Short 310 helix (residues Gly^33^ to Asn^35^)	2L86	[128]
Monomeric human hIAPP bound to cysteine free insulin-degrading enzyme (E111Q mutant)	X-ray diffraction	Monomeric hIAPP is embedded into several hydrophobic pockets of IDE, between residues 15 and 16 is one of the major cleavage sites. 2.9 Å resolution	3HGZ	[133]
Human, residues 20–29 SNNFGAILSS fragment, (core fibril domain)	Solid-state NMR	Antiparallel hetero zipper amyloid fibrils. 0.52 Å (backbone)/1.16 Å (heavy atoms) resolution	2KIB	[118]
Human, full-length monomeric IAPP, non-amidated, expressed in E. coli, bound to SDS micelles, pH 4.3	Solution NMR	Residues 1–4 form a hairpin loop by the single disulphide bond. Residues 5–28 form the α-helix core. Last nine residues are unfolded	2KB8	[32]
Human, IAPP NFLVHS segment	X-ray diffraction	Steric zipper, amyloid-like fibrils and microcrystals. 1.85 Å resolution	3FR1	[116]
Human IAPP AILSST segment	X-ray diffraction	Steric zipper, amyloid-like fibrils and microcrystals. 1.4Å resolution	3FOD	[116]
Human IAPP HSSNNF segment	X-ray diffraction	Steric zipper, amyloid-like fibrils and microcrystals. 1.5Å resolution	3FPO	[116]
Human, IAPP NVGSNTY form 1, hydrated crystal form	X-ray diffraction	Steric zipper, hydrated crystal form, protein fibril. 1.5Å resolution	3FTK	[116]
Human IAPP, NVGSNTY form 2, heptapeptide segment	X-ray diffraction	Dehydrated crystal form, classified as protein fibril. 1.6Å resolution	3FTL	[116]
Human IAPP, NFLVHSS segment	X-ray diffraction	Anti-parallel β-sheet with sulphate ion-bound. 1.84 Å resolution.	3FTH	[116]
Human SSTNVG, alternate polymorph, form 2	X-ray diffraction	Steric zipper, amyloid forming peptide, β-sheet packed face to face, 1.61 Å resolution	3FTR	[116]
Full-length human IAPP expressed in *E. coli*, fused to maltose-binding protein.	X-ray diffraction	Adopts an α-helical structure at residues 8-18 and 22–27. Molecules of IAPP dimerise, four chains, 1.86 Å resolution	3G7V	[117]
Human expressed in *E. coli*; Human/*E. coli* residues 1–22 fused to maltose-binding protein	X-ray diffraction	Adopts an α-helical structure. 1.75 Å resolution	3G7W	[117]
Human IAPP NNFGAIL (21-27) peptide	X-ray diffraction	Forms amyloid-like fibrils. 1.8 Å resolution	3DGJ	[115]
Human IAPP SSTNVG peptide (28–33)	X-ray diffraction	Form amyloid-like fibrils with cross-β spine. 1.66 Å resolution	3DG1	[115]
Residues 18–27 of hIAPP	Solid-state NMR, TEM and AFM	Forms a striated ribbon structure (protofilament) that contains four layers of parallel ß-sheets formed by two symmetric layers of IAPP molecules	N/A	[127]
An F15L/F23L/Y37L triple mutant (IAPP-3XL) absent of aromatic residues	AFM	Triple leucine mutant readily forms fibrils but at a lower rate compared with WT. Both have left-handed helix, both twisted ribbon morphology that's more pronounced in WT	N/A	[114]
Human, full-length peptide in complex with insulin-degrading enzyme	X-ray diffraction	Each IDE monomer comprises four homologous αβ roll domains. Forms β-sheets with IDE strands B12 and B6. 2.6 Å resolution	2G48	[132]
Human peptide (20–29) in membrane-mimicking environment	Solution NMR	hIAPP forms a distorted type I β-turn when it interacts with negatively charged micelles	1KUW	[113]

Data are taken from open-access, peer-reviewed publications and from data deposited in the Protein Data Bank (www.wwpdb.org) website. Abbreviations: DPC, dodecyl phosphocholine; EM, electron microscopy; EPR, electron paramagnetic resonance; Micro ED, micro electron diffraction; NMR, nuclear magnetic resonance; SDS, sodium dodecyl sulphate.

Structures have revealed that monomers tend to have a predominantly α-helical content (Table 2). The native monomeric form is considered a disordered unfolded protein with disulphide bonds between cysteine residues at positions 2 and 7 [137], however, NMR chemical shift studies demonstrated that the monomeric form contains a transient helical structure between residues 5 and 20 [138].

### Fibrillar structures

Fibrils tend to be characteristic of amyloid fibrils as seen in other PMDs usually being composed of two protofilaments and having a predominantly β-sheet content, (containing β-strands that run perpendicular to the fibre axis) that can be identified using X-ray fibre diffraction and cryo-electron microscopy (EM) (Table 2). Like other PMDs, more than one type of fibril (polymorph) was commonly observed from one sample preparation [123,125] adding to the complexity in understanding the structure of disease-associated hIAPP. The ability to isolate hIAPP fibrils in enough quantity to study their structure to high resolution has limited advances in obtaining their detailed structure, the same being true for smaller species such as monomers and oligomers hence the detailed structure of *bone fide* *ex-vivo* structures remain unknown.

A promising paper showed that hIAPP fibrils extracted from a T2DM patient could be used to seed synthetic hIAPP peptides to produce enough fibrils to study by cryo-EM [123]. Four different polymorphs were revealed, all showing twisted fibrils, composed of two intertwined protofilaments, three of which resemble the pathogenic seeds suggesting they may represent disease-related structures. These fibrils demonstrate typical amyloid structures being composed of layers of β-strands stacked with 4.8 Å spacing. Two of the polymorphs are composed of two conformationally distinct protofilaments (‘heterotypic fibrils’). A hydrogen bond associated with Tyr^37^ is consistent with the importance of amidation at the C-terminal thought to be important in fibril formation. The structures also explain one reason why the S20G hereditary mutation may facilitate hIAPP fibrillisation since glycine residues allow a more kinked conformation that in turn may allow the peptide chain to adopt a fibril-forming fold thus more easily forming a stable fibril structure. Since three of the polymorphs do not resemble any structures produced *in vitro* it suggests *in vitro* structures may differ from those *in vivo* as previously summarised for other amyloidogenic proteins [90] due to *ex-vivo* fibrils being more stable and protease-resistant which has important consequences for therapeutic design [139].

### Oligomeric structures

Oligomers have been less well studied. Some research suggests they have a more α-helical content [101], whilst others suggest a β-sheet content [102,130]. Oligomers composed of parallel β-sheets have been linked to the destruction and dysfunction of β-cells and other tissues [140]. Importantly, it has been suggested that interactions with membranes contribute to misfolding events and influence the structure of these hIAPP intermediates hence it may explain the differences in the observed structures produced.

Most of the structures solved to date are produced *in vitro* from peptides. Not all the structures are full-length, and the preparation methods used differ between studies. Hence, these factors may contribute to the different polymorphs produced as previously suggested [141] arguing that these structures may also not truly represent the structure of hIAPP *in vivo.*

**Figure 2:**
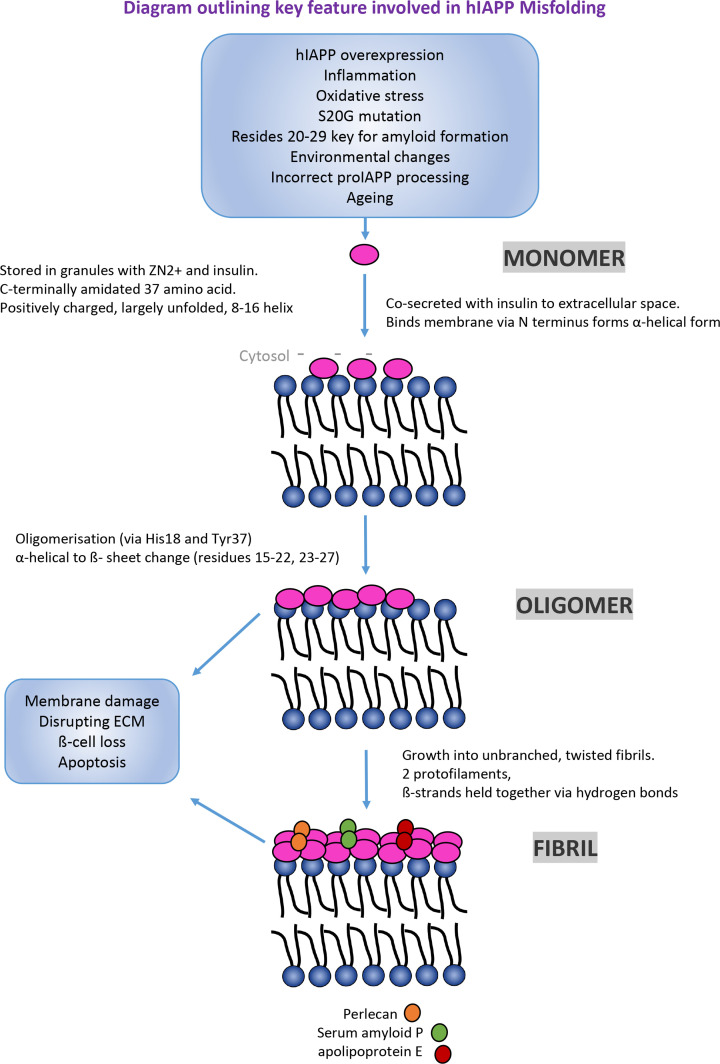
Cartoon diagram that highlights in the form of a simplified flow chart, the different protein conformations hIAPP adopts and how they relate to T2DM Outlined are the different factors thought to contribute at different stages of the misfolding process and key molecular features related to the different conformations.

We have summarised the current understanding behind the misfolding and aggregation of hIAPP and its link to T2DM in a simplified cartoon diagram shown in Figure 2.

### Residues in hIAPP responsible for amyloid formation

**Figure 3:**
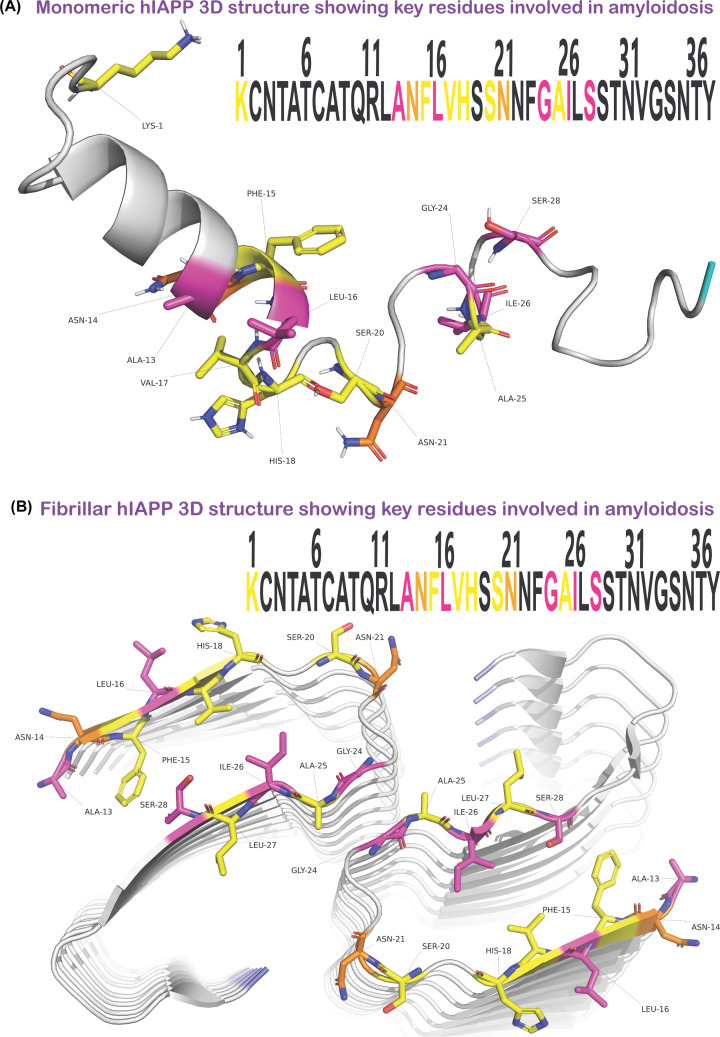
The identification of important residues linked to amyloidosis highlighted on solved 3D structures of hIAPP The colours highlight single residue mutations that abolish (purple), or accelerate (yellow) amyloid formation, or both (orange) and can be seen in the amino acid sequence within each figure. (**A**) Structure of recombinant hIAPP with an amidated C-terminal and oxidised cysteines, solved by solution NMR (PDB code = 5MGQ). (**B**) Structure of synthetic wildtype hIAPP peptide that is amidated at the C-terminal and aggregated *in vitro* to form fibrils and solved by cryo-EM (PDB code = 6Y1A). (A,B) are created using Pymol (http://www.pymol.org/pymol) and protein structures 5MGQ and 6Y1A were obtained from the Protein Data Bank (https://www.rcsb.org/).

Many mutational analysis experiments have been performed on hIAPP to understand the mechanism of hIAPP amyloid formation. To date, a large amount of data assessing the importance of certain residues in the sequence of hIAPP involved in amyloid formation have been collated. However, a systematic analysis of all possible mutations for positions of IAPP has not yet been published. Here we have summarised all mutational analysis studies that have been done to date using full-length (Supplementary File S1 and Table S1) or an 8–37 fragment of hIAPP (Supplementary File S1 and Table S2). Studies using shorter hIAPP fragments have been undertaken but are not included in this review as we deem them less physiologically relevant. Most of the studies summarised used hIAPP that is amidated at the C-terminal with point mutations altering a single amino acid at a time, to assess whether certain residues are involved in the ability of hIAPP to form amyloid (Supplementary Tables S1 and S2). For clarity, we have highlighted these residues in solved 3D structures of monomeric and fibrillar hIAPP (PDB codes: 5MGQ and 6Y1A), [142] as shown in Figure 3 created using Pymol [143]. These figures highlight single amino acids able to accelerate (shown in yellow) or abolish (shown in purple) amyloid formation, and in some cases do both (shown in orange). These important residues are located in the centre of the monomer structure (Figure 3A). The significance of their positioning is clearer in the disease-associated fibrillar form (Figure 3B). Most of the highlighted residues are present in the region covering the two β-stands and the connecting loop. Mutations such as S28P (Supplementary Figure S1) alone are able to abolish amyloid formation disrupting the formation of secondary structures thus preventing β-sheet formation. S20G is known to accelerate amyloid formation and this could be linked to the fact that it can form a new core fold within the protofilaments on to which a third protofilament can bind [124].

Mutating asparagine at position 14 can abolish (N14S, N14A, and N14L) or accelerate (N14D) amyloid formation. Hydrophobic residues (alanine and leucine) abolish amyloid formation whereas mutating to a negatively charged aspartic acid accelerates amyloid formation [144,145]. Similarly, mutations of N21 can also abolish (N21D, N21isoD, N21L, N21F, and N21S) and accelerate (N21A, N21P, N21n (d-amino acid)) amyloid formation of hIAPP. Understanding the molecular basis behind the link between different residues and amyloidosis clearly requires further exploration [144–146].

It should be noted that it is difficult to compare data between these different studies. This is due to different lengths of the protein being used (fragment vs full-length protein), the C-terminal amidation status differs and buffer type, buffer co-solutes, pH, ionic strength, and temperature used in the assays differ. In the truncated studies (Supplementary Table S2), even though the truncation is the first seven amino acids, outside the reported amyloid core, it may still affect the fibrillation rate and structures produced. Additionally, thioflavin-T binding assays used to assess hIAPP amyloid formation [147,148] used the same concentration of IAPP protein (16 µM) but used different concentrations of thioflavin-T (32 and 25 µM) and different buffer conditions [147,148]. This is important since the relative rate of amyloid formation can vary depending on the buffers being used [149]. The pH of the assay is also important since hIAPP fibrillation depends on the ionic strength and ion composition [150] and pH can affect the protonation status of the N-terminus and His^18^. The protonation of histidine in an acidic environment results in the prevention of cation–π interactions and the formation of hydrogen bonds with other polar or charged residues. Whereas in a neutral environment, deprotonation of His^18^ favours these interactions [150]. In addition, Thioflavin-T binding assays can give false negative and false positive results [149,151]. Hence, results should always be confirmed with other techniques such as EM or circular dichroism to confirm the presence of fibrils.

In summary, from the studies summarised in Supplementary Tables S1 and S2, various mutations in the SRE conserved region [20–29] of IAPP abolish amyloid formation, suggesting these residues as being important in amyloid formation and T2DM progression. Some species that do not show amyloid formation include dog, rat, mouse, guinea pig, degu, and cow [152]. Although all IAPP sequences from these species carry a S29P substitution, so does the IAPP for cat, which is also amyloidogenic. A triple substitution in hIAPP by proline (V17P/S19P/T30P), outside of the 20–29 region, has been shown to abolish amyloid formation [153]. More importantly, single residue substitutions outside of residues 20–29 are also able to abolish or significantly affect amyloid formation rate. This includes and is not limited to N14L, N14A, N14S, A13E, and L16Q (Supplementary Tables S1 and S2). None of these substitutions are present in non-amyloidogenic IAPPs of other species [144]. Clearly more extensive mutational studies are needed to fully understand the contribution of all the residues in the sequence to amyloid formation and toxicity.

## Interactions of IAPP with other amyloid forming proteins associated with disease

There are numerous reports that hIAPP can interact with other amyloidogenic proteins promoting amyloidosis, thus linking T2DM to other disorders such as AD [154]. This is thought to be possible when amyloidogenic proteins have structural and sequence similarities allowing them to cross-seed one another [155]. Recent cryo-EM structures comparing the structures of hIAPP and Aβ indeed reveal that Aβ PDB structures 60IZ show similarities with hIAPP 7M61 (chain A), Aβ 2M4J with hIAPP 7M61 (chain B) and 50QV and 6SHS matching with hIAPP 7M62 highlighting residues 19–29 of hIAPP and 24–34 Aβ as having structurally similar regions that may be responsible for cross-seeding [123]. *In vivo*, numerous studies have demonstrated that hIAPP can leave the pancreas and cross the blood–brain barrier and co-localise with Aβ in the brain contributing to the onset and progression of AD [41]. The similarity between the two diseases and the fact that an imbalance of glucose levels is present in AD brains has led to some researchers suggesting that AD should be renamed as ‘type 3 diabetes’ [156].

T2DM has been linked to PD. Recently, α-synuclein and IAPP have been shown to co-aggregate *in vitro* and *in vivo* in mice and human islet cells [76]. The fact that IAPP amyloid seeds promote α-synuclein amyloid formation *in vitro* could explain why having T2DM increases a person’s risk of getting PD but not the other way round since α-synuclein amyloid seeds actually inhibit IAPP amyloid production [157].

Another amyloidogenic protein, the prion protein, PrP (fragment 106–126) and IAPP are reported to co-aggregate which is interesting since they lack sequence homology [158]. Perhaps there is a link between T2DM and prion disorders, and perhaps other PMDs but this clearly needs to be explored further.

## Preventative measures and designing therapeutics for type 2 diabetes

In addition to understanding the structure of hIAPP to high resolution, understanding the mechanism of amyloid formation *in vivo* is needed for therapeutic design. Fibril formation is thought to involve a slow nucleation phase that may take many months or years as observed in other disorders such as AD [159] where individuals can remain asymptomatic for many years. This differs from what has been observed *in vitro* where hIAPP fibrils have been shown to occur in minutes [103].

Treatments for diabetes alleviate the symptoms but do not resolve the cause. Current treatments for diabetes include insulin, hIAPP analogues, biguanides, sulphonylureas, α-glucosidase inhibitors, dipeptidyl peptidase- 4 inhibitors, glucagon-like peptide receptor agonists, thiazolidinediones, SGLT2 inhibitors, and meglitinides [10].

### hIAPP analogues

Destruction of β-cells is linked to hIAPP and insulin deficiency observed in T1DM and T2DM [160,161], hence hIAPP analogues have been used in combination with insulin for the treatment of diabetes [162]. Only one analogue for hIAPP called Pramlintide, has been approved for clinical use [163]. Diabetes treatment with insulin can result in insulin-induced weight gain [164]. These side-effects can be treated with hIAPP analogues, which slow gastric emptying and reduce nutrient uptake, and have been shown to be effective as a dual treatment in combination with insulin [165]. hIAPP analogues such as Pramlintide were designed to reduce aggregation, but have some limitations. Although Pramlintide is soluble at acidic pH 4 it is less so at physiological pH, unlike insulin which is soluble at neutral pH. This means that the two cannot be stored together and must be given as two separate injections, which is not ideal hence better designed analogues are urgently needed.

Another analogue called Davalintide, not yet approved, binds to the amylin receptor [166]. However, it seems to bind irreversibly and is not as efficacious as Pramlintide plus it requires the use of an infusion pump to allow the slow release needed to induce significant weight loss [167]. Davalintide is a DACRA (Dual Amylin and Calcitonin Receptor Agonist) that can also activate calcitonin gene-related peptide receptors (CGRP-Rs) at high concentrations. The ability to activate both calcitonin and amylin receptors results in the prolonged activation of receptors thought to be more effective than amylin or calcitonin receptors alone.

KBP-088 is another DACRA and has been tested in clinical trials to treat weight loss and diabetes. Due to its high potency to bind both calcitonin and amylin receptors, it is a potent weight loss inducer. Using rats fed on a high-fat diet, those fed 5 μg/kg of KBP-088 per day for 11 weeks showed a 16% reduction in body weight [168]. However, five rats experienced excessive weight loss as a result of this treatment which is concerning and could be detrimental to health if administered to patients. A total of 10–15% of T2DM patients are not overweight and therefore KBP-088 treatment may not be the best treatment for them. In contrast, IAPP has been used as an adiposity signal for controlling weight and energy expenditure in animal studies [169,170] showing modest weight loss which could be a better alternative treatment for patients. Pramlintide given three times a day at a dose of 60 or 120–240 µg showed 1.7 and 3.7 kg weight loss in T1DM and T2DM patients over the course of 1 year respectively [171] equating to roughly 1–3% of total body weight compared with 16% weight loss in rats given KBP-088 [168]. Although these two studies are not directly comparable, the improved design of hIAPP analogues could be used to maintain weight loss after the initial use of a potent DACRA such as KBP-088.

Cagrilintide, which is a long-acting acylated IAPP analogue, is currently undergoing clinical trials for weight loss and glycaemic control in T2DM patients [172,173]. It is being used in combination with semaglutide, a glucagon-like peptide a (GLP1) agonist which enhances insulin release, to show an additive effect for weight loss as a treatment for obesity and T2DM [173]. Cagrilintide is also a DACRA binding to both amylin and calcitonin receptors [131]. Further research needs to be carried out to develop an IAPP analogue that binds amylin receptors preferentially over CTR like hIAPP, without being amyloidogenic.

Using hIAPP analogues in combination with other peptide hormones may accommodate the needs of individuals by mimicking the natural, energy homeostatic signalling pathways to create a specific desired physiological effect. For example, leptin, insulin, and peptide YY 3-36, a gut metabolic hormone, could be used in combination with an hIAPP analogue [174]. Clearly, more research is needed into better preventative and therapeutic treatments to target diabetes in the early stages before it causes multiple organ damage.

## Discussion

There are numerous studies that prove IAPP can form amyloid and a clear link between the accumulation of IAPP in T2DM patients and animal models strongly suggesting misfolded IAPP is linked to T2DM. The fact that only certain species (that have Ala^25^, Ser^28^, Ser^29^ in their IAPP sequence) form amyloidogenic forms of hIAPP and naturally get T2DM shows a direct link between the protein itself and T2DM.

There is extensive evidence linking dysfunction or loss of β-cells with reduced secretion of insulin and development of insulin resistance which is linked to IAPP amyloid formation [52]. However, the precise mechanisms involved remain unclear. IAPP amyloid formation is thought to occur via a nucleation-dependent polymerisation mechanism [88] intracellularly in the islet β-cell secretory granules then later released into the extracellular space [52]. What initiates these misfolding events is still unclear. Ageing may be a contributory factor since T2DM primarily affects adults. A disruption in proteostasis may induce intracellular aggregation of IAPP resulting in deposits of oligomers in the secretory pathway and granules. This could be linked to overexpression of hIAPP [61], which can lead to increased cytosolic Ca^2+^ activation of calpain and apoptosis [62], disruption of lysosomes and thus proteostasis [63], disruption of autophagy [64,65], and activation of the UPR. hIAPP aggregates have been shown to elicit inflammatory responses in β-cells directly or by recruiting macrophages [70–72]. The subsequent release of cytokines can induce insulin resistance in β-cells and secretion of IL-1β has been linked to IAPP aggregation [69]. Environmental factors such as oxidative stress may also contribute to IAPP aggregation as it has been observed that β-cells are particularly sensitive to oxidative stress [66].

As summarised in this review, evidence suggests that oligomers and fibrils are linked to T2DM (Figure 2) however like other PMDs there is still debate as to what conformation(s) are responsible for disease. Over the past decade, the number of structures of IAPP has dramatically increased (Table 2) due to technological advances (including better equipment and software) providing an insight into the protein itself and clues to how it may misfold. This includes mutational studies that have identified certain regions [20–29] important for amyloid formation (Figure 3, Supplementary Tables S1 and S2). There is ample evidence that demonstrates IAPP forms amyloid fibrils but the detailed structure of *ex vivo* fibrils still remains unknown. In addition, the *ex vivo/in vivo* structure of smaller conformations of IAPP that precede fibrils is unknown. Understanding the structure of these conformers is key to developing therapeutics since fibrillar conformers are linked to late stages of T2DM so being able to prevent or slow fibril formation may prevent onset of the disease. A recent study showed that the molecular chaperone Hsp70 binds to oligomers but not monomers [175] hence exploiting the use of chaperones as a therapeutic may be useful in the future as it only binds to misfolded forms. It is clear that interactions with membranes are an important factor in the misfolding events so understanding this interaction in more detail could also be an important target for therapeutics.

It appears that hIAPP can form more than one conformer of fibrils and oligomers (Table 2). This could be attributed to the array of different protein sources and fibrillisation conditions used for *in vitro* experiments. Using *ex-vivo* aggregates to seed fibrillisation reactions produced different conformations [123] suggesting that hIAPP may form more than one conformation in the body. Whether hIAPP can form different ‘strains’ as shown in other PMDs [176,177] is unclear but important to know when developing therapeutics since therapeutics may not work against all strains.

IAPP analogues such as Pramlintide, Davalintide, KBP-088, and Cagrilintide have all shown promise as a treatment for T2DM however issues with efficacy, solubility, and side effects need optimisation before they can be used as an effective T2DM treatment. Using natural compounds instead [139] or in combination with these analogues may alleviate some of the current issues including unwanted side effects.

More research is needed to identify what conformation hIAPP adopts when it deposits in different parts of the body (Table 1 and Figure 1) and in addition, identify other locations in the body where hIAPP accumulates. T2DM affects many different organs and can clearly be transported in the blood and is known to damage different parts of the body such as the heart, kidneys, eyes etc. Hence it does not seem unreasonable that it interacts with many different proteins in these organs and may also co-aggregate other amyloidogenic proteins accelerating the progression of other conditions. The ability of hIAPP to cross the blood–brain barrier is interesting. Importantly, since IAPP is able to bind to other amyloidogenic proteins such as amyloid-ß and α-synuclein [76,154], being able to stop misfolding of hIAPP may also prevent disease onset/progression of these other conditions. Understanding the precise molecular details involved in the misfolding of hIAPP may also reveal common mechanisms to other PMDs hence a pan-amyloid drug may be effective against more than one disease.

T2DM is clearly a complex disease. Interestingly, not all T2DM patients have hIAPP deposits [46] and hIAPP aggregates have been observed in asymptomatic healthy individuals [178,179] raising the question: what is the significance of these deposits and their relationship to T2DM, if at all? Are they the cause or consequence of the disease? Do they simply initiate or accelerate the condition, or do they form as a consequence when other regulatory systems fail? What other proteins does hIAPP bind to? Despite the first observations of IAPP aggregates from T2DM patients in 1901 [12] there is clearly still a long way to go to understand exactly how IAPP is linked to T2DM at the molecular level.

## Supplementary Material

Supplementary File S1

## Abbreviations

Aβ: amyloid-β
AD: Alzheimer’s disease
ATF6: Activating transcription factor 6
DACRA: Dual Amylin and Calcitonin Receptor Agonist
DN: diabetic nephropathy
EM: electron microscopy
HDAC NFAT: histone deacetylase nuclear factor of activated T cells
HDL: High-density lipoprotein
hIAPP: human islet amyloid polypeptide
HNE: 4-hydroxynonenal
IDE: insulin-degradation enzyme
IL-6: Interleukin 6
IL-8: Interleukin 8
IRF1: Interferon regulatory factor 1
MBP: Maltose Binding Protein
MCP-1: Monocyte chemoattractant protein-1
MDA: malondialdehyde
NLRP3: Nod-like receptor protein 3
PERK: Protein kinase RNA-like endoplasmic reticulum kinase
PMD: protein misfolding disorder
ROS: Reactive Oxygen Species
SRE: self-recognition element
T1DM: type 1 diabetes mellitus
T2DM: type 2 diabetes mellitus
TNF: Tumour necrosis factor
UPR: unfolded protein response
