# Supplementary material for: Linking hIAPP misfolding and aggregation with type 2 diabetes mellitus: a structural perspective
Source: Biosci Rep. 2022 May 18;42(5):BSR20211297. doi: 10.1042/BSR20211297 (PMC9118370; doi:10.1042/BSR20211297)
Supplement: Supplementary File S1 [file BSR-2021-1297C_supp.pdf]

Supplementary File S1

Table S.1: Summary of mutational studies using full-length synthetic hIAPP created with point mutations and their effects on amyloid formation, arranged with the most recent studies first.

**Mutation**<br>**Full length IAPP 1-37**	**Results**	**Citation**
A25T	Faster amyloid formation	(1)
K1I	Slightly slower amyloid formation	(1)
H18R	Slower amyloid formation	(1)
S28P S29P	Abolishes amyloid formation	(2)
A25P S29P	Slower amyloid formation	(2)
A25P S28P	Abolishes amyloid formation	(2)
S29P	Slower amyloid formation	(2)
S28P	Abolishes amyloid formation	(2)
A25P	Slower amyloid formation	(2)
Des L1 2-37 hIAPP	No effect reported	(3)
L1CML (carboxymethyl)-L-lysine	Faster amyloid formation	(4)
N21G	Slower amyloid formation	(5)
N21n (D-aminoacid)	Faster amyloid formation	(5)
N21Aib (aminoisobutyric acid)	Slower amyloid formation	(5)
N21P	Faster amyloid formation	(5)
N21Dab (diaminobutyric acid)	Abolishes amyloid formation	(5)
N21D	Abolishes amyloid formation	(5)
N21L	Abolishes amyloid formation	(5)
N21F	Abolishes amyloid formation	(5)
N21A	Faster amyloid formation	(5)
N31K	Slower amyloid formation	(6)
S29P	Slower amyloid formation	(6)
N22K	Slower amyloid formation	(6)
H18P	Slower amyloid formation	(6)
H18RS20G	Slower amyloid formation	(7)
S29P	Slower amyloid formation	(7)
S20G	Faster amyloid formation	(7)
H18R	Slower amyloid formation	(7)
H18E	Slower amyloid formation	(8)
H18A	Slower amyloid formation	(8)
H18K	Slower amyloid formation	(8)
H18R	Slower amyloid formation	(8)
N35isoD	No effect reported	(9)
N35D	No effect reported	(9)
N31isoD	Slower amyloid formation	(9)
N31D	Slower amyloid formation	(9)
N22isoD	slight slower amyloid formation	(9)
N22D	Slightly slower amyloid formation	(9)
N21D	Abolishes amyloid formation	(9)
N21isoD	Abolishes amyloid formation	(9)
N14isoD	No effect reported	(9)
N14D	Faster amyloid formation	(9)
Unamidated C terminal hIAPP	Slower amyloid formation	(10)
H18Q unamidated C terminal hIAPP	Faster amyloid formation	(10)
H18Q	Moderately faster amyloid formation	(10)
H18L	Faster amyloid formation	(10)
F15TLe (Tert-leucine)	Slower amyloid formation	(11)
F15I	Slower amyloid formation	(11)
F15NLe (Norleucine)	Slower amyloid formation	(11)

F15L F23L Y37L (3XL)	Slower amyloid formation	(11)
F23L Y37L	Slower amyloid formation	(11)
F15L Y37L	Slower amyloid formation	(11)
F15L F23L	Slower amyloid formation	(11)
Y37L	Slower amyloid formation	(11)
F23L	Slower amyloid formation	(11)
F15L	Faster amyloid formation	(11)
S20K	Slower amyloid formation	(12)
S20G	Faster amyloid formation	(12)
S28G	Abolishes amyloid formation	(13)
I26D	Abolishes amyloid formation	(13)
L16Q	Abolishes amyloid formation	(13)
A13E	Abolishes amyloid formation	(13)
H18R	Slower amyloid formation	(14)
G24P	Abolishes amyloid formation	(15,16)
I26P	Abolishes amyloid formation	(15,16)
F15L/F23L/Y37 3XL	Slower amyloid formation	(17)
A25P/S28P/S29P (Pramlintide)	Abolishes amyloid formation	(18)

Table S.2: Summary of all mutational studies using synthetic hIAPP fragment 8-37 and their effect on amyloid formation. The table is ordered in the reverse chronological order of references showing the most recent studies first.

**Mutation**<br>**Fragment of human IAPP 8-37**	**Results**	**Citation**
V17K	No effect reported	(19)
V17A	Faster amyloid formation	(19)
F15D	Faster amyloid formation	(19)
F15K	Slower amyloid formation	(19)
F15S	Faster amyloid formation	(19)
F15A	Faster amyloid formation	(19)
H18R/F23L/A25P/I26P	Abolished amyloid formation	(20)
H18R/S28P/S29P	Abolished amyloid formation	(20)
H18R/F23L	Slower amyloid formation	(20)
F23L	Slower amyloid formation	(20)
H18R	No effect reported	(20)
T36C	No change reported	(21)
T30C	Slower amyloid formation	(21)
A25C	Slower amyloid formation	(21)
V17C	Faster amyloid formation	(21)
A13C	Slower amyloid formation	(21)
N14L/L16N	Abolished amyloid formation	(21)
L12N/N14L	Abolished amyloid formation	(21)
N35L	Slower amyloid formation	(21)
N31L	Slower amyloid formation	(21)
N22L	No effect reported	(21)
N21S	Abolished amyloid formation	(21)
N21L	Abolished amyloid formation	(21)
N14S	Abolished amyloid formation	(21)
N14A	Abolished amyloid formation	(21)
N14L	Abolished amyloid formation	(21)
V17P/S19P/T30P (3XP)	Abolishes amyloid formation	(22)

References

1. Ridgway Z, Lee K-H, Zhyvoloup A, Wong A, Eldrid C, Hannaberry E, et al. Analysis of Baboon IAPP Provides Insight into Amyloidogenicity and Cytotoxicity of Human IAPP. Biophysical Journal. 2020 Mar;118(5):1142–51.

2. Ridgway Z, Eldrid C, Zhyvoloup A, Ben-Younis A, Noh D, Thalassinos K, et al. Analysis of Proline Substitutions Reveals the Plasticity and Sequence Sensitivity of Human IAPP Amyloidogenicity and Toxicity. Biochemistry. 2020 Feb 18;59(6):742–54.

3. Lee K-H, Zhyvoloup A, Raleigh D. Amyloidogenicity and cytotoxicity of des-Lys-1 human amylin provides insight into amylin self-assembly and highlights the difficulties of defining amyloidogenicity. Daggett V, editor. Protein Engineering, Design and Selection. 2019 Dec 13;32(2):87–93.

4. Hsu Y-H, Chen Y-W, Wu M-H, Tu L-H. Protein Glycation by Glyoxal Promotes Amyloid Formation by Islet Amyloid Polypeptide. Biophysical Journal. 2019 Jun;116(12):2304–13.

5. Godin E, Nguyen PT, Zottig X, Bourgault S. Identification of a hinge residue controlling islet amyloid polypeptide self-assembly and cytotoxicity. J Biol Chem. 2019 May 24;294(21):8452–63.

6. Akter R, Bower RL, Abedini A, Schmidt AM, Hay DL, Raleigh DP. Amyloidogenicity, Cytotoxicity, and Receptor Activity of Bovine Amylin: Implications for Xenobiotic Transplantation and the Design of Nontoxic Amylin Variants. ACS Chem Biol. 2018 Sep 21;13(9):2747–57.

7. Akter R, Abedini A, Ridgway Z, Zhang X, Kleinberg J, Schmidt AM, et al. Evolutionary Adaptation and Amyloid Formation: Does the Reduced Amyloidogenicity and Cytotoxicity of Ursine Amylin Contribute to the Metabolic Adaption of Bears and Polar Bears? Isr J Chem. 2017 Jul;57(7–8):750–61.

8. Khemtemourian L, Guillemain G, Foufelle F, Killian JA. Residue specific effects of human islet polypeptide amyloid on self-assembly and on cell toxicity. Biochimie. 2017 Nov;142:22–30.

9. Nguyen PT, Zottig X, Sebastiao M, Bourgault S. Role of Site-Specific Asparagine Deamidation in Islet Amyloid Polypeptide Amyloidogenesis: Key Contributions of Residues 14 and 21. Biochemistry. 2017 Jul 25;56(29):3808–17.

10. Tu L-H, Serrano AL, Zanni MT, Raleigh DP. Mutational Analysis of Preamyloid Intermediates: The Role of His-Tyr Interactions in Islet Amyloid Formation. Biophysical Journal. 2014 Apr;106(7):1520–7.

11. Tu L-H, Raleigh DP. The role of aromatic interactions in amyloid formation by islet amyloid polypeptide. Biochemistry. 2013 Jan 15;52(2):333–42.

12. Cao P, Tu L-H, Abedini A, Levsh O, Akter R, Patsalo V, et al. Sensitivity of Amyloid Formation by Human Islet Amyloid Polypeptide to Mutations at Residue-20. J Mol Biol. 2012 Aug 10;421(2–3):282–95.

13. Fox A, Snollaerts T, Errecart Casanova C, Calciano A, Nogaj LA, Moffet DA. Selection for Nonamyloidogenic Mutants of Islet Amyloid Polypeptide (IAPP) Identifies an Extended Region for Amyloidogenicity. Biochemistry. 2010 Sep 14;49(36):7783–9.

14. Wiltzius JJW, Landau M, Nelson R, Sawaya MR, Apostol MI, Goldschmidt L, et al. Molecular mechanisms for protein-encoded inheritance. Nat Struct Mol Biol. 2009 Sep;16(9):973–8.

15. Abedini A, Meng F, Raleigh DP. A Single-Point Mutation Converts the Highly Amyloidogenic Human Islet Amyloid Polypeptide into a Potent Fibrillization Inhibitor. J Am Chem Soc. 2007 Sep 1;129(37):11300–1.

16. Meng F, Raleigh DP, Abedini A. The Combination of Kinetically Selected Inhibitors in Trans Leads to the Highly Effective Inhibition of Amyloid Formation. J Am Chem Soc. 2010 Oct 20;132(41):14340–2.

17. Marek P, Abedini A, Song B, Kanungo M, Johnson ME, Gupta R, et al. Aromatic Interactions Are Not Required for Amyloid Fibril Formation by Islet Amyloid Polypeptide but Do Influence the Rate of Fibril Formation and Fibril Morphology. Biochemistry. 2007 Mar 1;46(11):3255–61.

18. Ratner RE, Dickey R, Fineman M, Maggs DG, Shen L, Strobel SA, et al. Amylin replacement with pramlintide as an adjunct to insulin therapy improves long-term glycaemic and weight control in Type 1 diabetes mellitus: a 1-year, randomized controlled trial. Diabet Med. 2004 Nov;21(11):1204–12.

19. Wiltzius JJW, Sievers SA, Sawaya MR, Eisenberg D. Atomic structures of IAPP (amylin) fusions suggest a mechanism for fibrillation and the role of insulin in the process: Mechanism of IAPP (Amylin) Fibrillation. Protein Science. 2009 Jul;18(7):1521–30.

20. Wiltzius JJW, Sievers SA, Sawaya MR, Cascio D, Popov D, Riekel C, et al. Atomic structure of the cross-β spine of islet amyloid polypeptide (amylin). Protein Science. 2008;17(9):1467–74.

21. Koo BW, Hebda JA, Miranker AD. Amide inequivalence in the fibrillar assembly of islet amyloid polypeptide. Protein Engineering, Design and Selection. 2008 Mar 1;21(3):147–54.

22. Abedini A, Raleigh DP. Destabilization of human IAPP amyloid fibrils by proline mutations outside of the putative amyloidogenic domain: is there a critical amyloidogenic domain in human IAPP? J Mol Biol. 2006 Jan 13;355(2):274–81.
